# The Effects of Augmented Reality Treadmill Walking on Cognitive Function, Body Composition, Physiological Responses, and Acceptability in Older Adults: A Randomized Controlled Trial

**DOI:** 10.3390/brainsci15080781

**Published:** 2025-07-23

**Authors:** Wei-Yang Huang, Huei-Wen Pan, Cheng-En Wu

**Affiliations:** 1 General Education Center, National Taiwan College of Performing Arts, Taipei 11464, Taiwan; pmp999@tcpa.edu.tw; 2 Office of Physical Education, National Cheng Kung University, Tainan City 701, Taiwan; p186598@gmail.com; 3 Office of Physical Education, Tamkang University, No. 151, Ying-Zhuan Rd., Tamshui, New Taipei City 251301, Taiwan

**Keywords:** augmented reality, cognitive function in older adults, virtual environment, the acceptance of AR technology, immersive experience

## Abstract

This study aimed to investigate the effects of augmented reality (AR) treadmill walking training on cognitive function, body composition, physiological responses, and acceptance among older adults. Additionally, it analyzed the relationships between body composition, physiological responses, and the acceptance of AR technology. A randomized controlled trial was conducted, recruiting 60 healthy older adults, who were assigned to either the experimental group (AR treadmill walking training) or the control group (traditional treadmill walking training). The assessments included cognitive function evaluation (stride length, walking speed, and balance test), body composition (BMI, skeletal muscle mass, fat mass, and body fat percentage), and physiological responses (heart rate, calorie expenditure, exercise duration, and distance covered). Furthermore, the AR Acceptance Scale was used to assess perceived ease of use, perceived usefulness, attitudes, and behavioral intentions. The results indicated that AR treadmill walking training had significant positive effects on improving cognitive function, optimizing body composition, and enhancing physiological responses among older adults. Compared with the traditional training group, the experimental group demonstrated better performance in stride length, walking speed, and balance tests, with increased skeletal muscle mass and reduced body fat percentage. Additionally, improvements were observed in heart rate regulation, calorie expenditure, exercise duration, and distance covered, reflecting enhanced exercise tolerance. Moreover, older adults exhibited a high level of acceptance toward AR technology, particularly in terms of attitudes and behavioral intentions, as well as perceived usefulness. This study provides empirical support for the application of AR technology in promoting elderly health and suggests that future research should explore personalized adaptation strategies and long-term effects to further expand the potential value of AR technology in elderly exercise.

## 1. Introduction

With the increasing trend of global population aging, promoting the physical health of older adults has become a central issue of concern in both academia and society [1,2]. Exercise not only helps improve physical function but also enhances mental health and overall quality of life [3,4]. It has profound effects on reducing the risk of chronic diseases, strengthening cardiopulmonary function, and improving overall life quality. In recent years, the development of digital technology has led to breakthroughs in the field of exercise and health. Among these, augmented reality (AR) technology has emerged as a potential tool for enhancing exercise engagement and effectiveness due to its highly immersive experience and interactivity [5,6]. Beyond exercise training, the application of AR technology in medical rehabilitation and health promotion has expanded, offering older adults innovative exercise models [7,8,9]. By integrating virtual environmental presentation and interactive functionalities, AR technology enables users to experience simulated outdoor walking, dynamic scene changes, and other elements during exercise, thereby increasing the enjoyment of physical activity [10].

Existing studies have preliminarily explored the application of augmented reality (AR) technology and highlighted its potential positive impact on cognitive function in older adults. However, the specific mechanisms by which AR treadmill walking training effectively enhances memory, attention, and executive function remain unresolved in current literature [11,12,13,14]. Furthermore, when examining the relationship between physical activity and cognitive function, research has indicated a close association between cognitive function and gait characteristics—including stride length, walking speed, and balance ability [15,16]. Notably, older adults experiencing cognitive decline often exhibit abnormal gait patterns [17], such as shortened stride length, reduced walking speed, and impaired balance control. These changes may further affect the independence of daily activities and overall quality of life, particularly increasing the risk of falls among older individuals [18]. Currently, discussions regarding the long-term effects of AR treadmill walking training and its broad applicability among older adults remain relatively limited. Therefore, this study aims to address this research gap by further investigating how AR treadmill walking training influences cognitive function in older adults while assessing its feasibility and practical value.

The acceptance of augmented reality (AR) technology among older adults requires further investigation, particularly in terms of perceived ease of use, perceived usefulness, and attitude and behavioral intention [19]. Perceived ease of use refers to the degree to which older adults find AR technology intuitive and manageable [20]. Perceived usefulness, on the other hand, represents their assessment of AR technology’s effectiveness in enhancing exercise performance and health management [21]. Attitude and behavioral intention encompass evaluations of AR technology’s role in improving exercise outcomes and reducing monotony, as well as the likelihood of continued use and recommendations to others [22]. These three dimensions serve as critical criteria for assessing AR technology acceptance in this study.

Additionally, AR technology has provided real-time feedback on physiological exercise data, such as heart rate monitoring, step counting, calorie consumption, exercise duration, and distance traveled [23]. This type of feedback helped users monitor their exercise status in real time, further enhancing their ability to manage physical activity [24]. For older adults, the application of this virtual technology reduced the monotony of exercise and improved its acceptability through visual and environmental stimulation [25]. However, existing studies still lack sufficient empirical support to verify the effects of AR technology on physiological exercise response data and technology acceptance among older individuals.

In summary, this study aimed to investigate the effects of AR treadmill walking training on the cognitive function of older adults, determine whether it optimized their body composition and physiological responses, and further analyze their acceptance of AR technology. Ultimately, the study evaluated the relationship between body composition, physiological responses, and the acceptance of AR technology. Based on these research objectives, this study explored the following key research questions: Did AR treadmill walking training improve the cognitive function of older adults? Did AR treadmill walking training optimize the body composition of older adults? Did AR treadmill walking training enhance the physiological responses of older adults? How did older adults perceive AR technology? What was the relationship between body composition, physiological responses, and AR technology acceptance among older adults?

To address these questions, the study proposed the following five research hypotheses:

**Hypothesis 1** **(H1).** *AR treadmill walking training had a significant impact on the cognitive function of older adults.*

**Hypothesis 2** **(H2).** *AR treadmill walking training had a significant impact on the body composition of older adults.*

**Hypothesis 3** **(H3).** *AR treadmill walking training had a significant impact on the physiological responses of older adults.*

**Hypothesis 4** **(H4).** *Older adults exhibited a high level of acceptance toward AR technology.*

**Hypothesis 5** **(H5).** *There was a significant correlation between body composition, physiological responses, and the acceptance of AR technology among older adults*.

## 2. Materials and Methods

### 2.1. Research Framework

**Figure 1:**
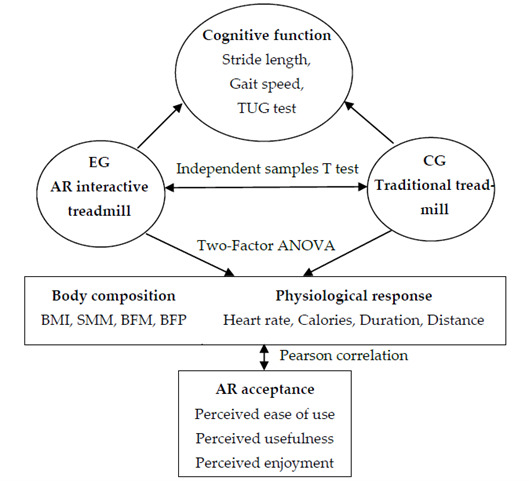
Research framework.

This study employed AR interactive treadmill walking training as the intervention method for the experimental group, and traditional treadmill training for the control group, to investigate intergroup differences in cognitive function, body composition, and physiological responses among older adults. Furthermore, the study analyzed the experimental group’s acceptance of and experience with AR technology. The overall research framework is detailed in Figure 1 to illustrate the logical relationships among the study design and variables.

### 2.2. Participants

This study targeted healthy older adults aged 65 and above in Taipei City as the population. Recruitment was conducted through posters at the fitness center of the academy, which included a QR code linking to registration information, online registration details, and printed registration brochures placed in the community service center. Posters were displayed on bulletin boards at the academy’s entrance, the community service center, and nearby convenience stores. The study publicly recruited community-dwelling older adults aged 65 and above who were capable of independent movement. Healthy older adults in this context were defined as individuals who could maintain normal functional abilities and general cognitive levels [26]. A total of 60 participants were recruited, meeting the required sample size [27].

**Figure 2:**
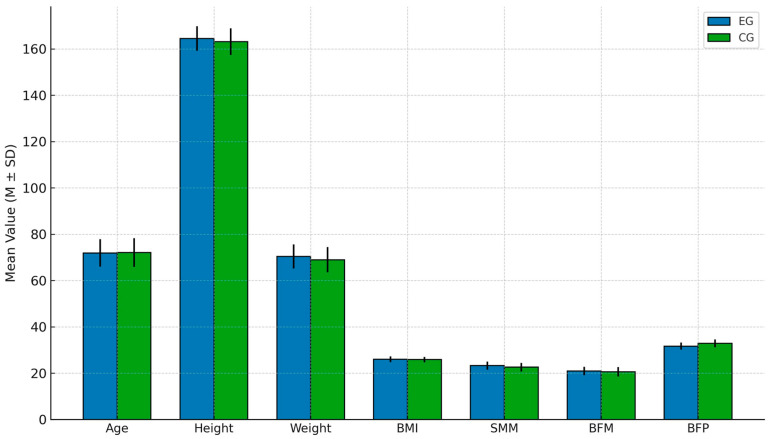
Homogeneity test of participants.

To ensure the representativeness of the sample, this study first employed the quantile–quantile plot (Q–Q Plot) and found that the sample exhibited normality [28]. The pre-test mean and standard deviation of the participants’ background variables showed a linear normal distribution, with values falling within the 95% confidence interval (CI) range [29]. Next, participants were evenly divided into an experimental group and a control group. A homogeneity test was conducted based on participants’ background variables, including age, height, weight, body mass index (BMI), skeletal muscle mass (SMM), body fat mass (BFM), and body fat percentage (BFP). The results indicated that there were no significant differences between the two groups (*p* < 0.05). The data presentation is shown in Table 1, and the homogeneity comparison of the two groups is illustrated in Figure 2.

All participants voluntarily participated and signed an informed consent form before the study began to ensure compliance with academic ethical guidelines. To guarantee the representativeness of the sample and the reproducibility of the research, the inclusion criteria required participants to be free from chronic diseases that affect mobility (e.g., cardiovascular disease or severe arthritis) and to have not engaged in regular AR-based exercise in the past three months. The exclusion criteria included individuals with severe visual or auditory impairments that could affect the AR experience. Additionally, participants who were unable to complete the required training and testing or failed to adhere to the study protocol were excluded.

### 2.3. Intervention

Participants engaged in training sessions three times per week, with each session lasting a total of 60 min. The sessions included a 10 min warm-up (dynamic stretching), 40 min of treadmill walking exercise, and a 10 min cool-down consisting of static stretching and relaxation. The intervention was conducted over a period of twelve weeks. This study adopted a 12-week training protocol, primarily from the perspective of physiological adaptation. Long-term training (12 weeks) typically elicited more stable and significant physiological adaptations, including improvements in aerobic endurance, muscle hypertrophy, neural transmission efficiency, and energy system regulation. Although short-term training (8 weeks) could produce initial effects, its adaptations in cardiovascular endurance, muscular strength, and explosive power were likely inferior to those resulting from the 12-week program [30].

**Figure 3:**
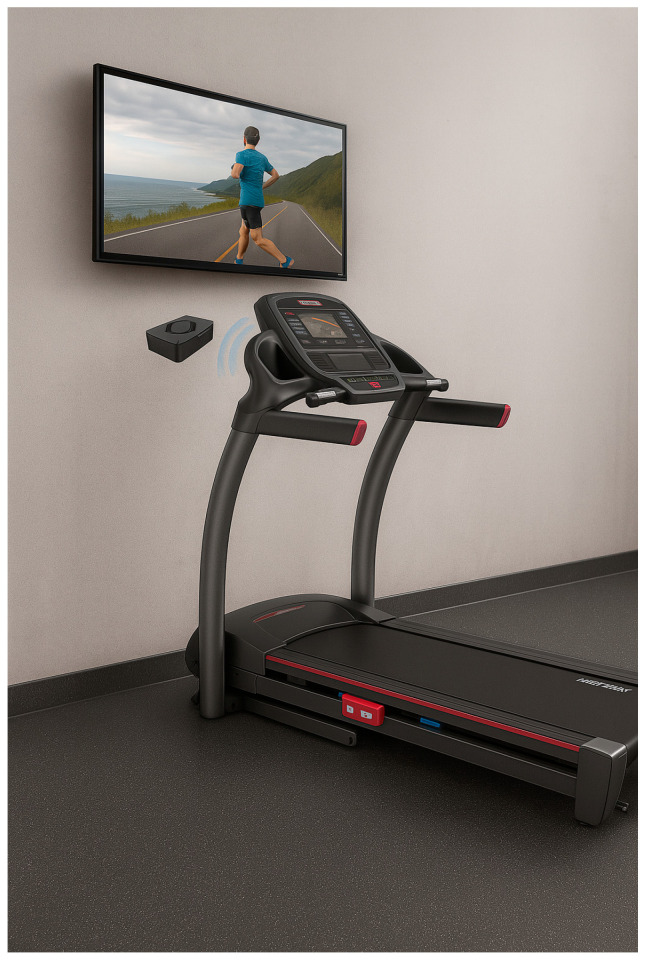
Combination of the AR interactive treadmill and Passport suite.

The experimental group in this study utilized “AR treadmill walking training” (AR simulated environment) [31]. The equipment used in this study was the JOHNSON 8.1T AR interactive treadmill (manufacturer: Johnson, Taichung, Taiwan), which included the Passport immersive interactive video system, as shown in Figure 3, which featured a built-in television and immersive interaction capabilities. This system featured four primary functions: 1. Three-Zone MaxComfort Adjustable Cushioning System: This system allowed a forward and backward displacement of 45 cm, enabling participants to adjust to the most comfortable running area and experience immersive environmental simulations (sand, forest trails, asphalt roads). 2. Interactive incline adjustment: The treadmill automatically adjusted the incline based on the terrain and road conditions encountered in the video, following the preset walking destination. 3. Speed synchronization with visuals: The virtual video playback speed adjusted in real-time to match the participant’s forward movement speed, ensuring a synchronized visual and physical experience. 4. fitVISTA Heads-Up Display: Various workout metrics were clearly displayed on the television screen, providing real-time data feedback.

The control group in this study utilized “traditional treadmill walking training” (without AR) [32]. The equipment used was the CHANSON electric treadmill (model: CS-6630, manufactured in New Taipei City, Taiwan). Unlike AR-equipped treadmills, this traditional treadmill lacked image-based interactive features. However, it was equipped with an IC interface display that recorded workout conditions.

### 2.4. Measurements

#### 2.4.1. Assessment of Exercise and Cognitive Function in Older Adults

This study aimed to investigate the effects of stride length, gait speed, and balance assessment (using the timed up and go test, TUG test) on participants’ cognitive function [33]. By analyzing pre-test and post-test variations in these exercise parameters, the study evaluated individuals’ physical activity capability. Although the aforementioned tests primarily reflected individuals’ sensorimotor functions, accumulated empirical research had indicated strong associations between gait parameters, balance control ability, and multiple cognitive processes, such as executive functioning, attention, and working memory. In particular, among older adults, declines in motor abilities frequently co-occurred with reductions in cognitive function, suggesting that such indicators could serve as early physiological signals for predicting or reflecting changes in cognitive status. Accordingly, this study adopted these assessments as indirect measures for evaluating changes in cognitive function and further verified their validity and representativeness through statistical analyses. Changes in these motor parameters were examined via pre- and post-test comparisons, with the specific assessment items described as follows.

1. Stride length: The 2-minute walk test (2MWT) was employed to estimate stride length (cm) by measuring walking distance and time [34]. The average stride length for healthy older adults is approximately 65 cm.
2. Gait speed: Participants walked 30 m at a normal pace, and the time required was recorded to calculate gait speed (m/s) [35]. The normal walking speed range for healthy older adults is 1.1–1.5 m/s; if the speed falls below 1.0 m/s, it may indicate a decline in physical activity ability.
3. Balance test (timed up and go test, TUG test): Participants stood up from a chair, walked 3 m, turned around, and returned to sit down, with completion time recorded [36]. If the test took more than 20 s, it could suggest balance issues.

#### 2.4.2. Pre- and Post-Test Measurement of Body Composition

Body composition was measured using an InBody 520 device (Biospace Co., Ltd., Seoul, Republic of Korea). The InBody 520 device estimated body composition based on bioelectrical impedance analysis (BIA) ([37]). Participants first entered their gender and age, stood on the InBody device, faced forward, and maintained an upright posture for approximately 60 s. Afterward, the body composition analysis results were obtained. This study collected pre- and post-test data for body mass index (BMI), skeletal muscle mass (SMM), body fat mass (BFM), and body fat percentage (BFP). According to Newman et al. [38], the appropriate BMI range for older adults is 20.0–26.9 kg/m^2^, while for very old adults aged 80 and above, a recommended range is 22.0–26.9 kg/m^2^. SMM tends to decline with aging; in general, male SMM accounts for 30–40% of body weight, while female SMM ranges from 25–35%. Older adults typically have higher BFM because muscle mass decreases while fat proportion increases with age. Additionally, BFP is generally higher in older adults compared with younger individuals; the healthy range for men is approximately 20–25%, while for women, it is around 25–30%.

#### 2.4.3. Assessment of Physiological Responses

Both the AR interactive treadmill and the traditional treadmill allowed for the monitoring of physiological indicators, including heart rate (real-time monitoring during exercise), calorie consumption, exercise duration, and distance covered.

According to this study, the standard physiological data ranges for healthy older adults during exercise were as follows [39]: 1. Exercise heart rate: Should be maintained at 50–70% of the maximum heart rate, calculated using the formula 220 − age. For example, the maximum heart rate of a 70-year-old individual is approximately 150 bpm, meaning the target heart rate range during exercise should be 75–105 bpm. 2. Calorie consumption during walking: Depends on body weight, exercise intensity, and duration. For instance, an older adult weighing 60 kg, walking at a speed of 4 km/h for 30 min, would burn approximately 105 kcal. 3. Recommended exercise duration: Older adults are advised to engage in 150 min of moderate-intensity exercise per week. 4. Walking distance: Determined by speed and duration. For example, at a walking speed of 5 km/h for 30 min, the distance covered would be approximately 2.5 km.

#### 2.4.4. AR Acceptance Scale

To measure individuals’ acceptance and usage of AR technology, this study referenced the Augmented Reality (AR) Acceptance Scale developed by Ustun et al. [40] and the motivation assessment for students using AR notes in classrooms proposed by Cabero-Almenara et al. [41]. The scale was refined and modified based on textual adjustments. The AR Acceptance Scale consisted of three dimensions: Perceived ease of use, perceived usefulness, and attitude and behavioral intention.

The scale included a total of nine items, all of which were positively framed. This study adopted a five-point Likert scale for measurement, with response options ranging from “Strongly Disagree” (1) to “Strongly Agree” (5). The highest agreement level (Strongly Agree) was assigned a score of 5, while the lowest agreement level (Strongly Disagree) was assigned a score of 1. Reliability analysis of the scale was conducted using Cronbach’s alpha. The Cronbach’s α values of the dimensions were all above 0.7, meeting the reliability standards [42]. The results indicated that the AR Acceptance Scale had a Cronbach’s α of 0.893, demonstrating consistency in reliability [43]. Validity analysis was performed using convergent validity, which measures the correlation and internal consistency of dimensions. The average variance extracted (AVE) values of the dimensions needed to reach 0.5 or higher to meet the validity criteria [44]. The results showed that the AVE value of AR acceptance was 0.774, indicating that the dimensions exhibited strong convergent validity.

### 2.5. Control Variables

To ensure the accuracy and comparability of the research results, this study established control variables across four aspects: 1. Participants: The study included healthy older adult males aged 65 to 80 years. All participants were equally divided into the experimental group and the control group, ensuring homogeneity to eliminate potential influences from age, gender, and health status on the results. 2. Use of augmented reality technology: The experimental group utilized the same AR treadmill, preventing technical variations from affecting the outcomes. 3. Experimental environment: All sessions were conducted in the same fitness center, ensuring that external environmental factors did not interfere with the results. 4. Psychological factors: Participants had no prior experience with AR treadmill-based exercise before the study, minimizing the impact of previous exposure or training on the results.

### 2.6. Statistical Analysis

First, descriptive statistics were used to quantify the background variables of the sample, presenting the data in terms of mean and standard deviation [45]. Next, a two-way repeated measures analysis of variance (ANOVA) was conducted to compare the interaction effects between body composition and pre- and post-tests in both groups. The effect size was examined using partial Eta squared (*η*^2^), where *η*^2^ > 0.14 indicated a large effect size [46]. For physiological indicators of the experimental group (AR treadmill) and control group (traditional treadmill), an independent samples *t*-test was performed, with the significance level set at *p* < 0.05. Additionally, descriptive statistics were applied to evaluate the AR Acceptance Scale within the experimental group.

Finally, the Pearson correlation coefficient was used to analyze the relationship between the AR Acceptance Scale and physiological indicators recorded during treadmill usage. The independent variable was AR acceptance, which included: perceived ease of use, perceived usefulness, and attitude and behavioral intention. The dependent variables consisted of physiological data, including: heart rate, calorie consumption, exercise duration, and distance covered.

## 3. Results

### 3.1. Effects on Cognitive Function in Older Adults

Both groups underwent pre- and post-test comparisons of stride length, gait speed, and TUG balance ability. Data analysis was conducted using two-way analysis of variance (ANOVA), and the results indicated significant improvements in both groups. However, the experimental group demonstrated F-values reaching significant levels, showing superior improvements compared with the control group. The specific findings in the experimental group were as follows: average stride length increased by 9.7% (*p* < 0.05, *η*^2^ = 0.84); average gait speed improved by 19% (*p* < 0.05, *η*^2^ = 0.92); and average balance ability (TUG test) increased by 21.7% (*p* < 0.05, *η*^2^ = 0.84). These three indicators showed high effect sizes, demonstrating notable improvements in cognitive-related motor abilities (see Table 2). Moreover, since the F-value represented the ratio of between-group variance (mean square between, MSB) to within-group variance (mean square within, MSW), defined as F = MSB/MSW, a higher F-value indicated that the between-group differences far exceeded the within-group differences—implying a strong effect of the independent variable on the dependent variable. The extremely high F-values in this study were attributed to two main factors: 1. Significant and consistent between-group differences: the independent variables and their interactions had a strong effect on the cognitive functions (dependent variable) of older adults, which led to a substantial increase in MSB and, consequently, a higher F-value. 2. Minimal within-group variance: the sample performance within each group was highly consistent, resulting in a very small MSW, which further amplified the F-value.

Further analysis indicated that the increase in stride length might be associated with improvements in neuromuscular control, while the enhancement in gait speed could be linked to greater walking efficiency among participants. Additionally, the improvement in TUG balance ability demonstrated significant gains in dynamic balance control, further validating the positive impact of this training method on overall gait and stability in older adults. Overall, the results of this study suggest that after AR treadmill walking training, the experimental group performed significantly better in cognitive function tests (stride length, gait speed, and balance) compared with the control group using traditional treadmill walking. This effect may be attributed to the real-time visual feedback and interactive challenges provided by AR technology, which encourage participants to engage in greater cognitive processing and decision-making during walking. This study confirmed H1: AR treadmill walking training has a significant effect on cognitive function in older adults.

### 3.2. Effects of Treadmill Walking Training on Body Composition in Older Adults

This study employed a controlled experimental design, where the experimental group underwent AR treadmill walking training while the control group used traditional treadmill training. After 12 weeks of training, a two-way analysis of variance (ANOVA) was conducted to compare the data. The results showed that F-values reached significant levels, indicating that the experimental group demonstrated greater improvements in body composition indicators compared with the control group. The specific findings were as follows: average weight reduction of 4.9% (*p* < 0.05, *η*^2^ = 0.91); average BMI decrease of 4.9% (*p* < 0.05, *η*^2^ = 0.92); average SMM increase of 4.3% (*p* < 0.05, *η*^2^ = 0.90); average BFM reduction of 11.3% (*p* < 0.05, *η*^2^ = 0.95); and average BFP decrease of 10.4% (*p* < 0.05, *η*^2^ = 0.85). These findings indicate that the body composition metrics in the experimental group displayed highly effective results (see Table 3). In addition, the elevated F-values were consistent with the explanation provided in Section 3.1.

Further analysis revealed that AR treadmill walking training not only had statistical significance but also demonstrated substantial benefits. The AR treadmill enhanced older adults’ engagement in exercise and may positively impact their overall health, further supporting the potential applications of AR technology in the field of exercise and health. This study confirmed H2: AR treadmill walking training has a significant impact on body composition in older adults.

### 3.3. Effects of Treadmill Walking Training on Physiological Responses in Older Adults

This study employed a controlled comparison between the experimental and control groups. The experimental group engaged in AR treadmill walking training, while the control group used traditional treadmill walking training. After 12 weeks of training, data analysis was conducted using an independent samples *t*-test, and the results indicated the following: the average heart rate of the experimental group was 4.2% higher than that of the control group (*p* < 0.05); the average calorie consumption in the experimental group was 10.1% greater than in the control group (*p* < 0.05); the average exercise duration of the experimental group exceeded the control group by 27.6% (*p* < 0.05); and the average distance covered in the experimental group was 37.1% greater than in the control group (*p* < 0.05).

These results demonstrate that physiological responses in AR treadmill walking training were superior to those in traditional treadmill walking training (see Table 4). Further analysis indicated that AR treadmill walking training enhanced exercise duration and distance, allowing older adults to sustain physical activity for longer periods. The increase in heart rate may contribute positively to exercise intensity and calorie consumption. These findings confirmed H3: AR treadmill walking training significantly influences physiological responses in older adults.

### 3.4. Analysis of the AR Acceptance Scale

This study employed the AR Acceptance Survey Scale, which consisted of nine items categorized into three factors: perceived usefulness, perceived ease of use, and attitude and behavioral intention. The survey results indicated that the AR Acceptance Scale in the experimental group showed significant differences in the F-test. Among the three factors, attitude and behavioral intention had the highest mean score (4.87), followed by perceived usefulness (4.75), while perceived ease of use was relatively lower, with a mean score of 4.59. Overall, the mean scores of all scale items ranged from 4.43 to 4.90, reflecting participants’ positive evaluation of AR technology in exercise applications (see Table 5).

Notably, with the F-values of the three factors showing significant differences, attitude and behavioral intention ranked highest in AR treadmill acceptance among participants, indicating a strong recognition of the engaging and motivational aspects of AR technology in exercise settings. This study confirmed H4: older adults exhibit a high level of acceptance toward AR technology.

### 3.5. Correlation Analysis Between Post-Test Body Composition, Physiological Responses, and AR Acceptance in the Experimental Group

In this study, to ensure data consistency and comparability, the body composition parameters (BMI, SMM, BFM, BFP), physiological data (heart rate, calorie consumption, exercise duration, and distance), and the three factors of the AR Acceptance Scale (perceived usefulness, perceived ease of use, and attitude and behavioral intention) were standardized using *Z*-score transformation. This process eliminated individual numerical differences, enhancing the accuracy of statistical analysis.

**Figure 4:**
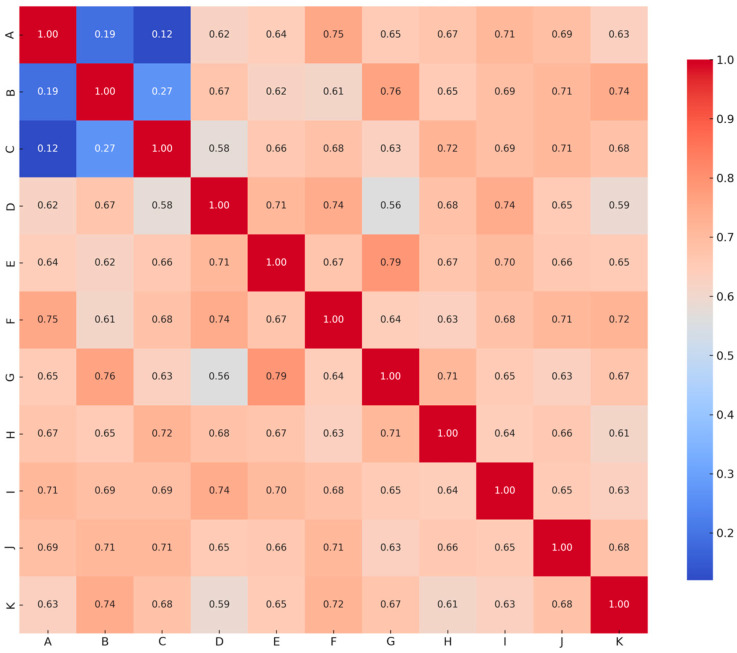
Correlation analysis between body composition, physiological, and AR acceptability. Note: Perceived ease of use is A, perceived usefulness is B, attitude and behavioral intention is C, BMI is D, SMM is E, BFM is F, BFP is G, heart rate is H, calories is I, duration is J, and distance is K.

Next, the Pearson correlation coefficient was applied to analyze the relationships between these variables (see Figure 4). The results indicated that while the three dimensions of the AR Acceptance Scale (perceived usefulness, perceived ease of use, and attitude and behavioral intention) exhibited a certain degree of correlation, their overall correlation coefficients were low, suggesting that these factors remained relatively independent in the participants’ evaluations.

However, when further examining the relationship between the AR Acceptance Scale and body composition and physiological data, it was found that all parameters showed a moderate to high positive correlation. These findings suggest that when participants engaged in AR treadmill walking training, they not only recognized the value and convenience of the technology on a psychological level but also demonstrated positive physiological effects. These results confirmed H5: the body composition and physiological responses of older adults using AR treadmill walking training are significantly correlated with AR technology acceptance.

## 4. Discussion

### 4.1. AR Treadmill Walking Training and Cognitive Function

The results of this study demonstrated that the experimental group significantly outperformed the control group in stride length, gait speed, and TUG balance ability, indicating that AR treadmill walking training had a positive impact on the gait and balance abilities of older adults [47]. This finding is consistent with previous research. For example, Jia et al. proposed that improving stride length and walking speed could reduce the risk of falls [48]. In addition, Yin et al. indicated that walking speed, stride length, and the results of the timed up and go (TUG) test were closely associated with the functional mobility of older adults [49].

The results of this study further support the impact of walking training on cognitive function in older adults. Jiménez-García et al. found that gait speed and balance ability are significantly associated with cognitive function in older adults, suggesting that better gait performance may be linked to enhanced cognitive abilities [15]. Moreover, AR treadmills can simulate various terrains and environmental conditions, allowing older adults to experience changes and challenges during exercise. Through virtual interactions, these treadmills provide diverse visual and dynamic stimuli, subtly capturing the attention of older adults and promoting sensory engagement [50]. This multi-sensory interaction not only enhances the enjoyment of walking but may also have positive effects on cognitive and neurological health. Han et al. pointed out that the use of AR technology can improve attention and memory in older adults, thereby fostering cognitive potential in daily life [51].

Moreover, the results showed that AR technology effectively enhances attention, aligns with personal interests, improves a sense of achievement, and facilitates exercise effectiveness in older adults, which is consistent with the findings of Quandt and Freitag [52]. Through vivid visual displays and interactive experiences, AR treadmills make exercise more engaging, further strengthening users’ focus and increasing their immersion in the activity. Additionally, the animated visuals provided by AR technology can enhance memory, thereby improving older adults’ cognitive responses to external stimuli, a phenomenon validated in the study by Goumopoulos et al. [53]. The application of AR technology not only fosters cognitive potential in daily life but may also have long-term positive effects on cognitive function through sustained use.

### 4.2. AR Treadmill Walking Training Improves Body Composition and Physiological Responses

In terms of physical health, Korn et al. reported that the application of AR technology has significant benefits for older adults [54]. The findings further revealed that participants using AR treadmills showed improvements in weight management, body mass index (BMI), skeletal muscle mass (SMM), body fat mass (BFM), and body fat percentage (BFP), supporting the potential of AR technology in elderly exercise interventions. Additionally, this study found that older adults rated their “attitude and behavioral intention” toward AR treadmill use the highest, primarily due to the immersive experience of the virtual environment, which enhanced the enjoyment of exercise.

Further analysis revealed a significant positive correlation between older adults’ acceptance of AR and physiological data (heart rate, calorie expenditure, exercise duration, and distance traveled). The findings indicate that AR treadmills help extend exercise duration, increase calorie consumption, and enhance walking distance, ultimately improving exercise tolerance. These results are consistent with previous studies [55,56]. Furthermore, recent research supports the application value of AR technology in elderly exercise interventions. For instance, a study on middle-aged and older adults with diabetes found that adjustments in appendicular lean mass (ALM), BMI, and body weight can effectively predict health risks and are closely related to exercise performance [57].

Another study found that moderate treadmill exercise significantly improved arterial oxygen saturation (SaO_2_), maximal oxygen uptake (VO_2_max), and walking distance in older adults, further supporting the positive impact of exercise on elderly health [58]. These findings are consistent with the results of this study, indicating that AR treadmills not only enhance body composition and physiological markers in older adults but also improve their exercise performance and overall health. This provides a new direction for future elderly exercise interventions.

### 4.3. Older Adults’ Acceptance of AR

The results of this study showed that older adults rated their acceptance of AR treadmills highest in terms of “attitude and behavioral intention,” reflecting that most participants believed AR technology could enhance the enjoyment of exercise, making it more engaging and providing an immersive workout experience. This immersive experience allows users to feel as if they are exercising in a natural environment, effectively promoting participation in physical activities [59]. On the other hand, the findings indicated that some older adults rated the “perceived usefulness” of AR technology the lowest, possibly due to the high learning curve associated with new technological products, requiring additional guidance and assistance. This may be related to cognitive decline, physical deterioration, and unfamiliarity, fear, or difficulty in understanding new technologies [60]. Therefore, in the promotion and design of AR treadmills, factors such as user-friendly interfaces and supportive mechanisms should be considered to lower adaptation barriers and enhance accessibility for older users.

Furthermore, the acceptance of AR treadmills is closely related to “perceived ease of use”. When users walk on the treadmill, they can freely adjust their walking speed, adapt to different terrains and slopes, and simulate a realistic exercise environment, thereby enhancing their sense of balance [61]. This adjustable exercise environment not only improves safety but also makes it easier for older adults to adapt and encourages continued use. Overall, this study confirmed that older adults exhibited a high acceptance of AR treadmills, aligning with the three key perceptual factors: perceived usefulness, perceived ease of use, and attitude and behavioral intention.

The results of this study showed that older adults generally exhibited high acceptance of AR technology. One of the key reasons is that AR systems can collect and store personal exercise data, effectively reducing memory load [62]. Older adults do not need to manually record their workout metrics, which helps alleviate frustration and anxiety associated with the learning curve of AR technology. For many older individuals, adopting new technology may present operational challenges. However, with the assistance of AR technology, the convenience of exercise experiences can be enhanced, further strengthening their willingness to participate consistently [63]. Additionally, during AR treadmill sessions, older adults can interact with the virtual environment, which naturally extends exercise duration and increases workout continuity [64]. Overall, this study confirmed that older adults demonstrated high acceptance of AR treadmills, primarily due to the automated management of exercise data and enhanced sensory stimulation. These findings provide strong support for the application of AR exercise technology in aging populations.

### 4.4. Future Prospects

The findings of this study demonstrated that AR technology holds significant potential for applications in elderly exercise interventions [65]. Future research and technological advancements may further optimize user interfaces and interaction mechanisms to enhance usability and expand the scope of application [66]. The outcomes of this study not only provide a crucial empirical foundation for the field of health technology but also offer valuable insights for the future development of assistive exercise technologies for older adults.

With continuous advancements in AR technology, its feasibility and accessibility have substantially improved [67]. In recent years, numerous studies have confirmed the effectiveness of AR technology in industries, education, and healthcare while also supporting its application value in sports and fitness [68,69,70]. Research has shown that older adults exhibit a high level of acceptance of AR technology, further highlighting its potential for development in elderly exercise interventions.

Future studies could focus on enhancing the introduction and training of AR technology to improve older adults’ ability and confidence in using these systems, thereby strengthening its application value in elderly physical activities. Additionally, further investigation into the impact of different AR designs on cognitive training, assessment of long-term training effects, and considerations of various exercise intensities, personalized adjustment plans, and prolonged use outcomes could help expand the empirical foundation of AR technology in elderly health promotion.

### 4.5. Research Limitations

Although this study provided an empirical foundation for the application of augmented reality (AR) technology in elderly exercise interventions, several limitations remain that need to be addressed in future research. First, the sample size was limited to 60 healthy older adults, which may affect the external validity of the results. Future studies could expand the sample size and include elderly populations with varying health conditions to enhance the applicability of the findings. Second, the intervention duration in this experiment was relatively short, and the long-term effects of training on cognitive function and body composition require further investigation. Future research could extend the training period to evaluate the physiological and psychological impacts of prolonged exercise. Additionally, this study considered only a specific AR interface design and did not compare the effects of different AR presentation formats on elderly exercise performance. Subsequent research could explore various AR interaction models to optimize user experience. Lastly, the acceptance of AR technology may be influenced by individuals’ digital literacy and prior experience with technology. Future studies could incorporate more social and psychological variables to comprehensively assess the potential of AR technology in elderly exercise applications.

## 5. Conclusions

This study examined the effects of augmented reality (AR) treadmill walking training on cognitive function, body composition, physiological responses, and acceptance in older adults, revealing the potential value of this technology in elderly exercise applications. The findings indicated that older adults who received AR treadmill walking training demonstrated improvements in cognitive function, particularly in stride length, gait speed, and balance tests, showing significantly greater progress compared with the traditional walking training group. Additionally, body composition analysis showed that the AR training group achieved better improvements in skeletal muscle mass (SMM) and body fat percentage (BFP), suggesting its potential positive impact on elderly health promotion. Regarding physiological responses, AR technology contributed to optimized heart rate regulation, increased calorie expenditure during walking, extended exercise duration, and improved walking distance, demonstrating its value in exercise assistance. Notably, older adults exhibited a generally high acceptance of AR technology, particularly in terms of attitude, behavioral intention, and perceived usefulness, further supporting its feasibility for aging populations. The results of this study not only provide empirical evidence for the application of AR technology in elderly health promotion but also offer guidance for future research and practical development. Future studies are recommended to explore personalized adaptation strategies and the long-term effects of AR interventions to expand their practical value in elderly exercise applications.

## Figures and Tables

**Table 1:** Homogeneity test of participants.

Variable	EG (*n* = 30) M ± SD	CG (*n* = 30) M ± SD	*t*-Value	*p*-Value
Age	71.9 ± 5.93	72.1 ± 6.23	−0.117	0.907
Height	164.5 ± 5.26	163.2 ± 5.75	1.050	0.303
Weight	70.4 ± 5.16	69.0 ± 5.45	0.975	0.338
BMI	26.0 ± 1.25	25.9 ± 1.18	0.321	0.751
SMM	23.3 ± 1.74	22.6 ± 1.86	1.356	0.185
BFM	20.9 ± 1.81	20.6 ± 2.06	0.565	0.576
BFP	31.7 ± 1.53	32.9 ± 1.67	−0.484	0.632

Note: EG represents the experimental group, and CG represents the control group. Body mass index is abbreviated as BMI, skeletal muscle mass is abbreviated as SMM, body fat mass is abbreviated as BFM, and body fat percentage is abbreviated as BFP. The mean ± standard deviation is expressed as M ± SD. The *t*-test values are represented as *t*-value (*p*-value).

**Table 2:** Two-factor analysis of variance for cognitive function.

Variable		EG (*n* = 30) M ± SD	CG (*n* = 30) M ± SD	F-Value Pre-Test and Post-Test	*η* ^2^	F-Value Pre- and Post-Tests * Two Groups	*η* ^2^
Stride length	Pre	59.43 ± 2.69	60.20 ± 3.15	305.40 *	0.84	147.62 *	0.72
	Post	65.20 ± 3.43	61.24 ± 3.18				
Gait speed	Pre	1.00 ± 0.09	0.99 ± 0.12	664.51 *	0.92	217.70 *	0.79
	Post	1.19 ± 0.11	1.04 ± 0.12				
TUG	Pre	12.81 ± 2.05	13.57 ± 1.56	296.62 *	0.84	166.97 *	0.74
	Post	10.03 ± 2.46	13.17 ± 1.58				

Note: The timed up and go was abbreviated as TUG. The mean ± standard deviation is expressed as M ± SD. The F values are represented as F-value. * *p* < 0.05.

**Table 3:** Two-factor analysis of variance for body composition.

Variable		EG (*n* = 30) M ± SD	CG (*n* = 30) M ± SD	F-Value Pre-Test and Post-Test	*η* ^2^	F-Value Pre- and Post-Tests * Two Groups	*η* ^2^
Weight	Pre	70.40 ± 5.16	68.99 ± 5.45	587.35 *	0.91	358.78 *	0.86
	Post	66.92 ± 4.89	68.56 ± 5.44				
BMI	Pre	25.99 ± 1.25	25.89 ± 1.18	680.11 *	0.92	409.58 *	0.88
	Post	24.71 ± 1.22	25.74 ± 1.19				
SMM	Pre	23.27 ± 1.74	22.65 ± 1.86	534.48 *	0.90	172.51 *	0.75
	Post	24.27 ± 1.86	22.92 ± 1.89				
BFM	Pre	20.93 ± 1.81	20.62 ± 2.06	1052.62 *	0.95	482.27 *	0.89
	Post	18.57 ± 1.74	20.16 ± 2.08				
BFP	Pre	31.65 ± 1.53	31.87 ± 1.67	340.17 *	0.85	210.61 *	0.78
	Post	28.35 ± 2.03	31.47 ± 1.77				

Note: EG represents the experimental group, and CG represents the control group. Body mass index is abbreviated as BMI, skeletal muscle mass is abbreviated as SMM, body fat mass is abbreviated as BFM, and body fat percentage is abbreviated as BFP. The mean ± standard deviation is expressed as M ± SD. The F values are represented as F-value. * *p* < 0.05.

**Table 4:** Independent samples *t*-test for physiological data.

Variable	EG (*n* = 30) M ± SD	CG (*n* = 30) M ± SD	*df*	*t*-Value	*p*-Value
Heart rate _(times)_	120 ± 6.45	115 ± 5.83	58	3.35 *	0.001
Calories _(kcal)_	209 ± 20.03	188 ± 21.07	58	4.04 *	0.000
Duration _(min)_	32.2 ± 2.70	23.4 ± 2.46	58	13.30 *	0.000
Distance _(km)_	2.59 ± 0.21	1.63 ± 0.26	58	15.77 *	0.000

Note: EG represents the experimental group, and CG represents the control group. The mean ± standard deviation is expressed as M ± SD. The *t*-test values are represented as *t*-value (*p*-value). * *p* < 0.05.

**Table 5:** Scale analysis.

Factors	Items	M ± SD	F	LSD
1. Perceived Usefulness	1. Using an AR treadmill enhances exercise performance.	4.43 ± 0.50	4.19 *	3 > 2 > 1
	2. Using an AR treadmill aids physiological monitoring.	4.80 ± 0.40		
	3. Using an AR treadmill provides physiological data.	4.83 ± 0.37		
	4. Using an AR treadmill increases exercise intensity demands.	4.30 ± 0.59		
2. Perceived Ease of Use	5. Using an AR treadmill is more convenient.	4.73 ± 0.44		
	6. Using an AR treadmill is easy to operate.	4.77 ± 0.42		
3. Attitude and Behavioral Intention	7. Using an AR treadmill motivates participation.	4.87 ± 0.34		
	8. Using an AR treadmill makes exercise enjoyable.	4.83 ± 0.37		
	9. Using an AR treadmill allows for both play and exercise.	4.90 ± 0.30		

Note: The mean ± standard deviation is expressed as M ± SD. * *p* < 0.05.

## Data Availability

Data on the participants were obtained before and after training. All authors confirm the authenticity and availability of the data. All datasets on which this paper’s conclusions are based have been made available to editors, reviewers, and readers.
